# Getting the Work-Nonwork Interface You Are Looking for: The Relevance of Work-Nonwork Boundary Management Fit

**DOI:** 10.3389/fpsyg.2018.01158

**Published:** 2018-07-17

**Authors:** Yanne Bogaerts, Rein De Cooman, Sara De Gieter

**Affiliations:** ^1^Department of Work and Organisation Studies, University of Leuven, Leuven, Belgium; ^2^Research Group of Work & Organizational Psychology, Vrije Universiteit Brussel, Brussels, Belgium

**Keywords:** work-nonwork interface, boundary management, person-environment fit, work-life conflict, well-being

## Abstract

Recently, work-family scholars have empirically demonstrated the importance of congruence between employees' boundary management preferences and boundary management supplies provided by the work environment in relation to employee attitudes and behavior. However, a theoretically grounded construct that captures this congruence is lacking. The present study addresses this gap by developing the construct and measure of *work-nonwork boundary management fit*, based on the needs-supplies fit framework. We cross-validate the scale in three independent samples (*n* = 188, diverse group of employees, *n* = 75, employees from one hospital, and *n* = 81, employees from one car company) and in a fourth sample (*n* = 458, working parents), we demonstrated the importance of work-nonwork boundary management fit for employee well-being (i.e., stress and work-life conflict). In particular, we confirmed its unique role in predicting employee well-being, above and beyond workload and work interrupting nonwork behaviors. Hence, we argue for considering work-nonwork boundary management fit when studying how work-family policies and organizational culture affect employees in the workplace.

## Introduction

Research devoted to the interplay of work and nonwork (e.g., family and leisure) life continues to flourish, as “balancing” both roles becomes a vital concern to an increasing proportion of employees that engages in multiple roles (Kelly et al., 2008; Allen and Martin, 2017). Work–life conflict or an interrole conflict resulting from the incompatibility of work and nonwork demands is a common source of stress and is found to be detrimental to employees' health and family functioning, as well as their workplace functioning (Amstad et al., 2011). Finding a fulfilling work and private life and being successful in both domains has thus become a critical factor and an important struggle for individual well-being (Grzywacz and Carlson, 2007; Allen and Martin, 2017). Hence, many employees in today's workforce are facing the challenge of managing the boundaries around their work and nonwork roles in a way that promotes positive outcomes in their work, family, and personal life (Capitano et al., 2017).

How employees manage work and nonwork boundaries is primarily described as a matter of free choice (Nippert-Eng, 1996). However, scholars pointed out that someone's personal and idiosyncratic preference for integrating or separating work and nonwork roles (i.e., boundary management preference) cannot always be enacted, due to constraints and expectations from the work environment (Ammons, 2013; Rothbard and Ollier-malaterre, 2016). The impact of fit between an employee's boundary management preference and organizational boundary management supplies (i.e., an organizational culture and formal policies that foster segmentation or integration) has recently received increased scholarly attention (e.g., Kreiner, 2006; Chen et al., 2009; Rothbard and Ollier-malaterre, 2016). Theories on needs-supplies fit highlight that individuals long to be in organizations that match their personal characteristics and therefore express more positive attitudes and behavior at work when their personal needs are more fully addressed by their work environment (Kristof-Brown and Guay, 2011). Personal needs originate from the desired amount of an attribute and fit exists when an employee's environment provides the resources required to satisfy his/her personal needs (Edwards and Rothbard, 1999). The desired amount of segmentation or integration between work and nonwork roles represents such a personal need on which fit perceptions can apply. Therefore, fulfilling one's boundary management need by providing a “fitting” work environment (e.g., work-family policies and culture) can thus contribute to achieving a more harmonious work-nonwork interface.

Several studies confirmed that a work environment that fits an employee's boundary management preference results in higher levels of job satisfaction and organizational commitment (Rothbard et al., 2005), improved mental health (Edwards and Rothbard, 1999), and reduced work-life conflict and stress (Kreiner, 2006; Chen et al., 2009). Although these studies make a strong case for the importance of person-environment fit, we argue that our understanding of this particular form of interaction is incomplete without considering how an employee's personal experience of fit in this regard affects outcomes. These prior studies measured fit as the calculated congruence between an employee's personal boundary management preference and perceived boundary management supplies. Yet, various fit scholars argue that it is the fit as actually felt and perceived by the employee that affects him or her. Empirical evidence indeed demonstrates that perceptions of fit are better predictors of employee attitudes, behavior, and well-being than the mere congruence between personal preferences and environmental supplies (Cable and Derue, 2002; Kristof-Brown et al., 2005). Therefore, there is a need to develop a theoretically grounded construct that captures employees' personal experience of fit between their personal boundary management preference and the boundary management supplies provided by their work environment.

To that end, we (1) introduce the new construct and corresponding measure of *work-nonwork boundary management fit* and (2) demonstrate its relevance by establishing the relation with employee well-being.

In doing so, this study contributes to the work-family literature by introducing a sound new theoretical construct that offers insights into the perception of congruence between an employee and his/her work environment with regard to boundary management. This is relevant since work-family studies have consistently shown that family-friendly policies and a culture of integration or segmentation are not universally beneficial to all employees; their effectiveness seems to depend on various individual differences (e.g., Rothbard et al., 2005; Foucreault et al., 2016). Nevertheless, these studies were not able to truly clarify individual differences in effectiveness. Applying a needs-supplies fit perspective to the work-nonwork interface does allow us to grasp these individuals differences and helps us to understand why and when a certain work environment is stressful and harmful for some employees, but not for others. In addition, in contrast to the growing body of research devoted to the calculated fit between separate measures of boundary management preference and workplace boundary management supplies (e.g., Rothbard et al., 2005; Kreiner, 2006), this study focusses on the specific construct of perceived fit, a portrayal of fit that is arguably more fundamental and proximal to affect, cognition and behavior (Cable and Derue, 2002). Thus, studying perceived fit in boundary management has the potential to significantly add to the contribution work-family scholars make toward improving employee well-being and organizational effectiveness. We not only introduce the concept of work-nonwork boundary management fit, we also developed, tested, and validated a corresponding scale that allowed us to demonstrate the added value of work-nonwork boundary management fit in relation to employee well-being.

## Theoretical foundations

### Managing work-nonwork boundaries

Boundary theory provides a theoretical framework for understanding how people manage the boundaries between their work and nonwork roles (Nippert-Eng, 1996; Ashforth et al., 2000; Rothbard et al., 2005). A core tenet of this theory is that individuals actively develop boundaries around both their work and personal life domains that vary in strength. Strong boundaries between work and nonwork domains are created in order to maintain work and nonwork as distinct domains, for example by having separate e-mail accounts, calendars, and key rings. Likewise, blurred boundaries allow these domains to be intermingled, for example by displaying family pictures at work and socializing with colleagues after work (Bulger et al., 2007). These strategies for managing work-nonwork boundaries fall on a continuum from segmentation (i.e., strong boundaries) to integration (i.e., blurred boundaries) (Ashforth et al., 2000).

The impact of boundary management strategies on the work-nonwork interface has received significant scholarly attention (Allen et al., 2014). Despite this growth in academic interest, there is some disagreement about whether having high levels of either integration or segmentation is helpful for balancing multiple roles or may actually lead to more conflict (Kelly et al., 2008; McNall et al., 2015). Over the past few years, scholars have argued that integrating or segmenting work and nonwork roles is not inherently good or bad, but, that the consequences of the boundary management behavior rather depend on the interaction between personal (i.e., boundary management preference) and organizational (i.e., boundary management supplies) factors (Kreiner, 2006; Chen et al., 2009; Foucreault et al., 2016). Organizational factors are found to have a considerable influence on employees' ability to act on their boundary management preference (Rothbard et al., 2005; Foucreault et al., 2016). Employees are exposed to an organizational culture (i.e., practices, norms, and expectations) and formal policies that may promote or discourage work-nonwork segmentation or integration, regardless of their boundary management preference. Consequently, employees may enact particular strategies of boundary management that are incongruent with their own boundary management preference, because of organizational constraints and perceived social expectations (Rothbard et al., 2005; Ammons, 2013). Indeed, previous studies found that an organizational culture and formal practices that foster integration (e.g., flextime, taking work home, and replying to colleagues' emails in the evening) appeal to employees desiring integration, but make employees desiring segmentation feel less committed to their organization and less satisfied with their job (Rau and Hyland, 2002; Rothbard et al., 2005). Overall, a workplace environment that fits an employee's boundary management preference is found to contribute to reduced work-life conflict and stress (Kreiner, 2006; Chen et al., 2009) and improved job satisfaction, organizational commitment (Rothbard et al., 2005), and mental health (Edwards and Rothbard, 1999). Although these studies provide evidence for the importance of fit in boundary management preference and supplies, recent developments in the person-environment fit literature suggest that researchers need to refocus their attention beyond the calculated interaction of separately measured personal and environmental factors involved in the striving for a certain work-family interface, and consider employees' personal experience of fit (Kristof-Brown and Billsberry, 2013).

### Person-environment fit theory

P-E fit is broadly defined as the congruence that occurs when employees and organizations are well matched (Kristof-Brown and Guay, 2011). It is based on the notion that personal and environmental characteristics do not only directly affect individual outcomes, but that human behavior is a function of both the person and the environment (DeRue and Morgeson, 2007; Kristof-Brown and Guay, 2011). Therefore, individuals have the tendency to seek out and create “fitting” environments that allow them to manifest their personality (Su et al., 2014). Although many types of fit have been identified, fit with the organization and fit with the job are the two most commonly examined forms (Kristof-Brown et al., 2005). From an organizational perspective, employees must fit both the job and the organization as a whole in order to be successful, thus making person-job and person-organization fit the main focus of recruitment, selection, and socialization practices (Cable and Derue, 2002). In this study, we focus on needs-supplies fit or whether the job and/or organization supplies what the employee needs in terms of work-nonwork boundary management. According to needs-supplies fit theory, the relation between fit and employee outcomes is grounded in the idea that fit leads to more positive work attitudes, behavior, and well-being through need satisfaction (e.g., Kristof-Brown, 1996; Cable and Edwards, 2004; Greguras and Diefendorff, 2009). Theories on psychological need satisfaction are based on the underlying process of cognitive comparison of needs and desires and the supplies provided by the environment (Cable and Edwards, 2004). Needs refer to biological and psychological requirements, often related to values and motives, whereas supplies consist of resources and rewards that may fulfill a person's needs (Cable and Edwards, 2004). A “fitting” environment thus affords employees the opportunity to satisfy their personal needs, leading them to attribute positive emotions to that environment and act to benefit that environment (Boon et al., 2011). A lack of fit signifies insufficient or inappropriate supplies and therefore unfulfilled needs. This lack of fulfillment creates stress, thereby reducing individual well-being (Edwards and Rothbard, 1999).

Kristof-Brown and Billsberry (2013) identified two dominant and inherently different portrayals of fit. Calculated fit looks at the interplay of separately measured personal and environmental factors to determine whether or not there is a match, while perceived fit taps into an individual's sense of “fitting in” by explicitly asking individuals to report their perceptions about how well they fit in Kristof-Brown and Billsberry (2013). Perceived fit thus captures fit as the psychological experience of congruence. Primarily the experience of fit, rather than the calculated congruence between person and environment, is found to drive attitudes and behavior of individuals in the workplace (Cable and Derue, 2002). Notably, this experience is more proximal to employees' decision making and behavior because needs-supplies fit may serve as a personal resource only if employees consciously experience it (Mackey et al., 2017). Despite its significance, perceived fit has nevertheless attracted comparatively little research and is therefore a promising area for new organizational fit research (Kristof-Brown and Billsberry, 2013).

### Work-nonwork boundary management fit

Drawing on the basic tenets of needs-supplies fit, we posit that an employee's preference for a certain degree of segmentation or integration of work and nonwork life is an individual need on which fit perceptions are based. Work-nonwork boundary management fit is thus defined as *an employee's psychological experience of congruence between his/her personal boundary management preference and the boundary management supplies of his/her work environment*. The experience of work-nonwork boundary management fit derives from the underlying process of cognitive comparison of an employee's need for integration (or segmentation) and perceived boundary management supplies as provided by the workplace. In particular, employees who feel that their boundary management preference—regardless of whether it leans toward segmentation or integration—is met by the culture and policies in the workplace are expected to experience high levels of work-nonwork boundary management fit. Conversely, when employees feel that their work environment does not meet their boundary management needs, they may perceive low levels of work-nonwork boundary management fit. Hence, employees in the same work environment may therefore experience a different level of fit. In this respect, Ammons (2013) pointed out that although employees make some changes to their enacted boundaries, their broad boundary preferences were relatively stable over time. Qualitative findings from Billsberry et al. (2008) and Chuang et al. (2015) support the assumption that fit perceptions can be based on employees' boundary management preference. Using a qualitative approach, they explored the underlying meaning of employees' sense of fit and identified work-life balance (whereof boundary management makes an important part) to be a key “fit-theme” that emerged from employees' own experiences of fit.

We thus assume that the perception of work-nonwork boundary management fit contributes to achieving a harmonious work-nonwork interface. Employees who feel that their boundary management needs are met by their work environment have access to organizational resources (e.g., formal policies and expectations) that allow them to create boundaries according to their preference. A fitting work environment allows employees to manage their work and nonwork demands within their preferred boundaries and establish their desired level of work-nonwork interference. This facilitates the reconciliation of work and nonwork demands and empowers employees to create their ideal work-nonwork interface, thereby preventing work-life conflicts to occur (Kreiner, 2006). Furthermore, a fitting work environment is sought after and valued because perceptions of fit serve as a personal resource associated with favorable outcomes for both employee and organization (Mackey et al., 2017). Employees in a “fitting” work environment feel reinforced as an individual, which leads them to attribute positive emotions to the organization, feel more involved with the organization, contribute to the organization in constructive ways, and feel a strong bond with the organization making leaving less attractive (Cable and Derue, 2002; Kristof-Brown and Guay, 2011).

## Materials and methods

### Overview of the studies

To develop and evaluate the work-nonwork boundary management fit scale, we followed a multiphase process (e.g., DeVellis, 1991; Hinkin, 1998; Worthington and Whittaker, 2006). First, a review of relevant literature was used to develop a detailed conceptual definition of work-nonwork boundary management fit. Thereafter, we created a measure for work-nonwork boundary management fit based upon existing needs-supplies fit measures. Next, we explored the factor structure in a first study. In a second study, we performed a confirmatory factor analysis and evaluated the internal consistency reliability of the measure. Subsequently, we evaluated the construct validity (convergent and discriminant) and criterion-related validity of the work-nonwork boundary management fit scale in the third study. In the fourth study, we aimed to demonstrate the relevance and impact of work-nonwork boundary management fit by examining the relation with employee well-being. Participation in all studies was voluntary and anonymous. We did not obtain a formal ethical committee approval for this study as our university's ethical committee considered the proposed research design to be non-invasive and harmless. An overview of the studies can be found in Table 1.

**Table 1:** Overview of the studies.

	**Sample**	***n***	**Aim**
Study 1	Snowball sampling	188	EFA
Study 2	Hospital employees	75	CFA
Study 3	Car company employees	81	Construct validity *Convergent* *Discriminant* Criterion-related validity
Study 4	Employed parents	458	Stepwise hierarchical regression

*All surveys were administered in Dutch*.

### Scale development

We constructed the work-nonwork boundary management fit scale based upon two existing scales measuring perceived needs-supplies fit with both the job and the organization (Cable and Derue, 2002; Vogel and Feldman, 2009). The measure was restrained to four items with the intention of minimizing similarity between the items. Formulating more items would also increase the risk that these items would capture other (unintended) forms of fit (e.g., value congruence), whereas the work-nonwork boundary management fit scale is supposed to exclusively measure needs-supplies fit. Subject matter experts who have conducted research and published in work-family and P-E fit domains evaluated the quality and clarity of these items. They evaluated the item wording and the extent to which each item assessed the intended construct. Items were reworded for clarity based on their feedback. The final measure thus exists of four items including: “*My need for combining work and private life is met by the opportunities offered by my organization,” “In terms of the way I want to combine work and private life, this organization fits me well,” “In terms of the way I want to separate work and private life, this job fits me well,”* and “*My need for separating work and private life is met by the culture and habits in my organization.”* These items are scored on a seven-point Likert scale ranging from strongly disagree (1) to strongly agree (7).

### Study 1

The aim of the first study was to initially evaluate the factor structure of the work-nonwork boundary management fit scale. We assed inter-item correlations, Kaiser and scree-test, the amount of variance explained and the item factor loadings to explore the scale's factor structure.

#### Sample

Participants were recruited through an online social network using snowball sampling and participation was voluntary and anonymous. Inclusion criteria were (1) being at least 18 years and (2) having a paid job. A total of 188 employees participated in the study. Respondents' mean age was 41 years (*SD* = 11.74) and 35% were male. Seventy-three percent of the respondents worked fulltime. In terms of occupation, 73% of the respondents were white-collar workers, 12% were self-employed, 10% were civil servants, and 5% were blue-collar workers. Eighty-four percent of the respondents had a cohabiting partner and our respondents had one child on average (*SD* = 1.38). The sample was diverse, yet dominated by white-collar employees.

#### Results

Maximum likelihood estimation, without specifying the number of factors, was used to explore the factor structure. As recommended by Hinkin (1998), a number of criteria should be met in an exploratory factor analysis. First, all inter-item correlations should be over 0.40. Items that show an intercorrelation below 0.40 can be eliminated. Second, the number of factors that emerge on both the Kaiser (i.e., eigenvalues >1) and the screen test should equal the number of scales being developed. Third, the total percentage of variance explained should be high, with a minimum of 60% variance explained. Finally, all items should have a loading over 0.40 on the appropriate factor. An examination of inter-item correlations showed high intercorrelations (see Table 2) between the four items of the work-nonwork boundary management fit scale (ranging from 0.58 to 0.73), indicating that no item should be dropped. Both the screen test and the number of factors with an eigenvalue >1 suggested retaining one factor. The factor explained 74.14% of the variance, with an eigenvalue of 2.97. Finally, as shown in Table 3, each item loaded highly on the intended factor, with an average of 0.81. In sum, based on the item retention criteria, no items should be dropped or required modification. EFA showed that all items loaded highly on one factor and the factor adequately explained the total item variance.

**Table 2:** Study 1: Inter-item correlations.

	**Item 1**	**Item 2**	**Item 3**	**Item 4**
1. My need for combining work and private life is met by the opportunities offered by my organization	–			
2. In terms of the way I want to combine work and private life, this organization fits me well	0.62^***^	–		
3. In terms of the way I want to separate work and private life, this job fits me well	0.61^***^	0.73^***^	–	
4. My need for separating work and private life is met by the culture and habits in my organization	0.73^***^	0.66^***^	0.58^***^	–

*n = 188*;

*** *p < 0.001*.

**Table 3:** Item statistics and standardized factor loadings.

	**Study 1**			**Study 2**			**Study 3**			**Study 4**		
	***n*** = **188**			***n*** = **75**			***n*** = **81**			***n*** = **458**		
	**Mean**	***SD***	**Factor loading**	**Mean**	***SD***	**Factor loading**	**Mean**	***SD***	**Factor loading**	**Mean**	***SD***	**Factor loading**
Item 1	4.90	1.44	0.81	5.29	1.25	0.95	4.49	1.41	0.74	4.57	1.65	0.82
Item 2	4.90	1.48	0.83	5.39	1.25	0.90	4.56	1.41	0.83	4.72	1.56	0.85
Item 3	5.03	1.49	0.79	5.35	1.23	0.81	4.78	1.41	0.83	4.90	1.56	0.77
Item 4	4.76	1.46	0.82	5.23	1.20	0.93	4.23	1.54	0.75	4.52	1.61	0.74
Scale	4.89	1.27		5.31	114		4.51	1.21		4.68	1.36	

*Item scoring ranged from strongly disagree (1) to strongly agree (7). All factor loadings are significant at the 0.001 level*.

### Study 2

Further, continuing to follow the guidelines for scale validation of Hinkin (1998) and Worthington and Whittaker (2006), we needed to confirm the factor structure and assess reliability of the work-nonwork boundary management fit measure. To this aim, we collected additional data.

#### Sample

Participants in this study were hospital employees, who were invited to participate by the head of their department. Seventy-five employees completed the survey, resulting in a response rate of 37.5%. Respondents' mean age was 41 years (*SD* = 11.53), and their average tenure was 11 years (*SD* = 11.03). Sixty percent of the respondents were male and 75% worked fulltime. Seventy-nine percent of the respondents had a cohabiting partner and respondents had one child on average (*SD* = 1.02).

#### Results

A confirmatory factor analysis was conducted on the four work-nonwork boundary management fit items to confirm the assumption that a single factor underlies these items. The one-factor model of work-nonwork boundary fit fitted the data very well: χ^2^_(6)_ = 289.57, *p* < 0.05, *CFI* = 1.000, *TLI* = 1.008, *RMSEA* = 0.000, *SRMR* = 0.009. *SRMR* values close to 0.08 or below and *RMSEA* values close to 0.06 or below indicate acceptable model fit, with smaller values indicating a better model fit, whereas *CFI* and *TLI* values larger than 0.90 indicate good fit, and values larger than 0.95 indicate excellent fit (Brown and Cudeck, 1993; Hu and Bentler, 1999). Therefore, these fit indices satisfy the cut-off levels and indicate excellent model fit. All items loaded highly and significantly on the intended factor, with an average of 0.90. For specific factor loadings and item statistics, see Table 3. The work-nonwork boundary management fit scale exhibited a Cronbach's alpha reliability of 0.94, which exceeded the conventionally accepted minimum of 0.70 and was taken to be internally consistent (Hinkin, 1998).

### Study 3

The goal of the third study was to examine the construct and criterion-related validity of the work-nonwork boundary management fit scale. Construct validity, or the extent to which an operationalization measures the concept it is supposed to measure, was assessed by evaluating the relation between work-nonwork boundary management fit and measures designed to assess similar constructs (i.e., convergent validity) and dissimilar measures (i.e., discriminant validity; Bagozzi et al., 1991). To evaluate convergent validity, we used a measure of calculated fit between segmentation preference and workplace segmentation supplies. Correlations between perceived work-nonwork boundary management fit and calculated fit between segmentation preference and supplies were expected to be positive, moderate, and significant, as work-nonwork boundary management fit derives from the cognitive comparison of fit between preference and workplace supplies in boundary management. Furthermore, discriminant validity refers to the extent to which measures of different concepts are distinct (Bagozzi et al., 1991). To evaluate discriminant validity, we examined the relation between work-nonwork boundary management fit and task variety, a theoretically distinct variable. Task variety refers to “the degree to which a job requires employees to perform a wide range of tasks on the job” (Morgeson and Humphrey, 2006, p. 1,323). Work-nonwork boundary management fit is theoretically distinct from the variety of tasks of the job itself and task variety is known to be an important variable in predicting individual outcomes (Morgeson and Humphrey, 2006).

Lastly, we evaluated criterion-related validity by establishing the relation with theoretically related outcome variables. Work-nonwork boundary management fit may facilitate the achievement of a harmonious work-life interface because employees have access to an organizational culture and formal policies that fit their preference, allowing them to create their ideal work-life interface. Therefore, we expect that work-nonwork boundary management fit positively relates to work-life balance and negatively to work-life conflict. In addition, based on the P-E fit theory tenet that fit results in positive attitudes toward the organization, we expect work-nonwork boundary management fit to be positively related to job satisfaction and organizational commitment and negatively to turnover intention.

#### Sample

This sample included employees from one local Belgian branch of a multinational car company. Data were collected through a call for participation of the HR department. One hundred and eighty employees received an invitation to participate in a work-life survey. A total of 81 employees returned the survey, which translated into an effective response rate of 45%. Most of the employees were male (65%), with a mean age of 42 years (*SD* = 11.72). Seventy-six percent of the employees had a cohabiting partner and had 2 children on average (*SD* = 1.14) living in their household. Ninety-one percent of the respondents worked fulltime and their average tenure was 12 years (*SD* = 10.02).

#### Measures

The variables in this study were measured using 7-point Likert scales. The anchors for the scales were 1 (strongly disagree) and 7 (strongly agree). All items were translated into Dutch and we performed a back-translation to acquire accurate translations.

##### Calculated fit between boundary management preference and workplace supplies

We used eight items of Kreiner (2006) to capture personal segmentation preference and organizational segmentation supplies and calculated fit between them using absolute difference scores. An example of the preference items is “I don't like to have to think about work when I'm at home.” The corresponding workplace item is “My workplace lets people forget about work when they're at home.” Cronbach's alpha for segmentation preference and segmentation supplies were, respectively 0.86 and 0.90.

##### Task variety

We measured task variety by using four items of Morgeson and Humphrey (2006). Sample items were “The job requires a variety of skills” and “The job requires me to utilize a variety of different skills in order to complete the work.” Cronbach's alpha for this scale was 0.93.

##### Work-life conflict

We used six items developed by Carlson et al. (2000) to measure work-life conflict, focusing on time-based and strain-based conflict. Sample items are “My work keeps me from my family activities more than I would like” and “I am often so emotionally drained when I get home from work that it prevents me from contributing to my family.” Cronbach's alpha for this scale was 0.90.

##### Work-life balance

Work-life balance was measured using six items of Carlson et al. (2009). Sample items were “I am able to accomplish the expectations that my supervisors and my family have for me” and “People who are close to me would say that I do a good job of balancing work and family.” Cronbach's alpha for this scale was 0.81.

##### Job satisfaction

Job satisfaction was evaluated using Camman et al.'s (1979) three-item subscale from the Michigan Organizational Assessment Questionnaire. Sample items were “All in all I am satisfied with my job” and “In general, I like working here.” Cronbach's alpha for this scale was 0.87.

##### Organizational commitment

Organizational commitment was measured with the six-item subscale of affective organizational commitment developed by Meyer et al. (1993). Participants were asked to indicate the extent to which they agreed with items such as “I do feel like part of the family at my organization” and “This organization has a great deal of personal meaning for me.” Cronbach's alpha for this scale was 0.87.

##### Turnover intention

Turnover intention was measured by Camman et al. (1979) three-item scale. Sample items were “I often think of leaving the organization” and “It is very possible that I will look for a new job within the next year.” Cronbach's alpha for this scale was 0.73.

#### Results

Preliminary support for convergent validity was found given that all items loaded highly and significantly on the latent variable, with an average of 0.79 (see Table 3) and the Cronbach's alpha coefficient indicated good internal scale reliability and consistency (α = 0.87). The average amount of variance extracted (AVE) for our construct exceeded 0.50, suggesting that the items accounted for more truth than error in our construct (Brocato et al., 2012). In addition, convergent validity was evaluated by examining correlations among established measures of theoretically relevant variables and work-nonwork boundary management fit. The correlation between work-nonwork boundary management fit and the calculated fit between preference and supplies was *r* = 0.37 (*p* < 0.01), showing convergent validity while underlining that both scales are not measuring the same concept. These findings are in line with prior studies examining the relation between measures of perceived and calculated fit, showing similar correlations ranging from 0.08 to 0.41 (Cable and Judge, 1997; Kristof-Brown and Guay, 2011). Support for discriminant validity was found given that work-nonwork boundary management fit was statistically unrelated (*r* = −0.02, ns.) to task variety, a theoretically distinct variable. Overall, work-nonwork boundary management fit is related to but distinct from theoretically relevant variables, providing general support for convergent and discriminant validity. All correlation coefficients can be found in Table 4.

**Table 4:**
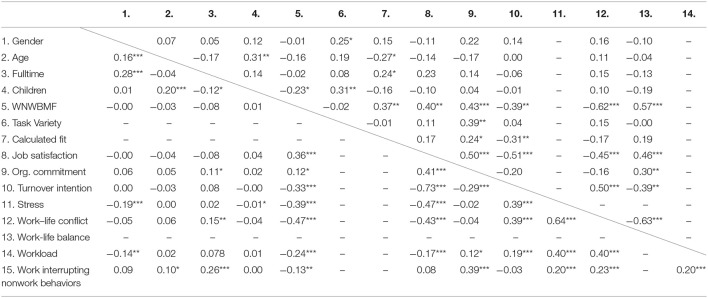
Pearson correlations Study 3 and Study 4.

Pearson correlations of Study 3 can be found above the diagonal, n = 81. Pearson correlations of Study 4 can be found under the diagonal, n = 458. Gender: 1 = Male, 0 = Female. Working fulltime: 1 = Fulltime, 0 = Part-time. ^*^p < 0.05. ^**^p < 0.01. ^***^p < 0.001.

Criterion-related validity of work-nonwork boundary management fit was evaluated by examining the relation with relevant outcome variables. We found support for criterion-related validity given the positive relation with job satisfaction (*r* = 0.40, *p* < 0.01), organizational commitment (*r* = 0.43, *p* < 0.001), and work-life balance (*r* = 0.57, *p* < 0.001), and the negative relation with turnover intention (*r* = −0.39, *p* < 0.01) and work-life conflict (*r* = −0.62, *p* < 0.001). According to Cohen (1988), the effect sizes in our study vary from moderate (*r* > 0.30) to strong (*r* > 0.50). Therefore, the assumed relationships attain statistical significance and thus provide evidence for criterion-related validity (Hinkin, 1998). All correlation coefficients can be found in Table 4.

### Study 4

In our final study, we aimed to demonstrate the relevance of work-nonwork boundary management fit by establishing the relation with employee well-being. Employees that experience work-nonwork boundary management fit are provided with resources that match their personal needs, thereby preventing work-life conflicts. Furthermore, needs-supplies fit may act as a personal resource (Demerouti et al., 2001) that provides stress-resistance potential because employees in a fitting work environment are better able to cope with organizational demands and stressors (Mackey et al., 2017). Previous studies have already identified workload and allowing work to interfere with the private life to be important predictors of stress and work-life conflict (e.g., O'Driscoll et al., 1992; Major et al., 2002; Olson-Buchanan and Boswell, 2006; Carlson et al., 2015). Notably absent from these models is cognitive appraisal, which is key in understanding why a certain environment is stressful for some people, but not for others. Stress and conflict derive not from personal or environment characteristics separately, but, rather, derive from a perceived mismatch between the environment and the person's needs (Edwards and Rothbard, 1999). Work-nonwork boundary management fit directly incorporates this cognitive appraisal. Therefore, we posit that the experience of work-nonwork boundary management fit contributes uniquely to the experience of stress and work-life conflict. Support for the unique contribution of work-nonwork boundary management fit can be found by demonstrating that it accounts for a significant amount of variance in work-life conflict and stress, beyond the variance explained by workload and integration behaviors.

#### Sample

This sample included working parents with at least one child under the age of 12. The pool of participants was obtained through Belgian childcare institutions, kindergartens and primary schools which invited parents to participate. A total of 458 individuals returned self-report surveys, resulting in a response rate of 9%. Eighty-five percent of the respondents were female, with an average age of 37 years (*SD* = 4.97). The majority of the respondents (92%) had a cohabiting partner and respondents had two children on average (*SD* = 0.80) living in their household. The majority of our participants are white-collar workers (94%) who represent a variety of job levels including entry level workers (48%), middle management and professionals (35%), and managers (11%). Fifty-eight percent of the respondents worked fulltime.

#### Measures

The variables in this study were measured using 7-point Likert scales. The anchors for the scales were strongly disagree (1) and strongly agree (7). All items were translated into Dutch and we performed a back-translation to acquire accurate translations. The Cronbach's alpha coefficient of work-nonwork boundary management fit indicated good internal scale reliability and consistency (α = 0.85). The scale evaluating work-life conflict (α = 0.88) was identical to that in Study 3.

##### Occupational stress

Stress was measured by Anderson et al.'s (2002) seven-item scale. Participants were asked to indicate the extent to which they had certain experiences during the past 3 months. Example items were “Have you felt emotionally drained from your work?” and “Have you felt used up at the end of the workday?” Respondents were asked to indicate how often they had felt each of seven emotions in the past 3 months, ranging from never (1) to always (5). The Cronbach's alpha for occupational stress was 0.89.

##### Workload

Workload was assessed with three items from the Dutch version (Furda, 1995) of Karasek's (1985) Job Content questionnaire. The scale included items that refer to quantitative and demanding aspects of the job (e.g., working hard, having too much work to do). Sample items were “How often does it happen that you have to much work to do?” and “How often does it happen that you have to work very fast?” The Cronbach's alpha for this scale was 0.86.

##### Work interrupting nonwork behaviors

Work interrupting nonwork was measured using Kossek et al.'s (2012) cross-role interruption behaviors scale of 5 items. Sample items were “I regularly bring work home” and “I work during my vacations.” The Cronbach's alpha for work interrupting nonwork behaviors was 0.76.

#### Results

Separate three-step, hierarchical regression analyses were performed for each outcome variable in order to test the unique role of work-nonwork boundary management fit in predicting stress and work-life conflict. Correlations can be found in Table 4. Hierarchical regression analysis allows for assessing the unique contribution of a variable in explaining variation in the independent variable (Cohen and Cohen, 1983). Results of these analyses can be found in Table 5. At step 1, we entered four control variables: gender, number of children, working full- or part-time, and working hours. These variables need to be controlled for, given their general potential to inflate or suppress relations between dependent and independent variables. Number of children and gender were included because they have been meta-analytically associated with work-life conflict (e.g., Aryee et al., 2005; Byron, 2005). Additionally, we controlled for working full- or part time and effective working hours given their relation with work-life conflict and stress (e.g., Major et al., 2002). Our results showed that female employees experience more stress (β = −0.22, *p* < 0.001) and work-life conflict (β = −0.12, *p* < 0.05) compared to male employees. Working hours also significantly predicted work-life conflict (β = 0.17, *p* < 0.01). At step 2, workload and work interrupting nonwork behaviors were entered as predictors. Both workload and work interrupting nonwork behaviors were significant predictors of stress (β = 0.35, *p* < 0.001; β = 0.15, *p* < 0.01) and work-life conflict (β = 0.36, *p* < 0.001; β = 0.13, *p* < 0.01). Workload tended to be a more important predictor of stress and work-life conflict, compared to work interrupting nonwork behaviors. Finally, we entered work-nonwork boundary management fit as predictor in step 3. Work-nonwork boundary management fit significantly predicted stress (β = −0.31, *p* < 0.001) and work-life conflict (β = −0.39, *p* < 0.001), beyond workload and work interrupting nonwork behaviors. Support for our hypothesis was found given that work-nonwork boundary management fit accounted for unique variance associated with stress (Δ*R*^2^ = 0.09, *p* < 0.001) and work-life conflict (Δ*R*^2^ = 0.14, *p* < 0.001) above and beyond the variance already explained by workload and work interrupting nonwork behaviors.

**Table 5:** Hierarchical regression coefficients.

	**Stress**	**Work-life conflict**
**Step 1**	β	β
Gender	−0.22^***^	−0.12^*^
Number of children	0.08^+^	−0.01
Working fulltime	0.01	0.06
Working hours	0.11	0.17^**^
*R*^2^	0.05	0.04
**Step 2**		
*Gender*	−0.14^**^	−0.04
*Number of children*	−0.10^*^	−0.03
*Working fulltime*	−0.00	0.06
*Working hours*	−0.03	0.05
Workload	0.35^***^	0.36^***^
Work interrupting nonwork behaviors	0.15^**^	0.13^**^
*R*^2^	0.21	0.19
Δ*R*^2^	0.15^***^	0.15^***^
**Step 3**		
*Gender*	−0.15^**^	−0.05
*Number of children*	−0.10^*^	−0.03
*Working fulltime*	−0.01	0.04
*Working hours*	−0.02	0.05
*Workload*	0.28^***^	0.27^***^
*Work interrupting nonwork behaviors*	0.13^**^	0.10^**^
Work-nonwork boundary management fit	−0.31^***^	−0.39^***^
*R*^2^	0.30	0.33
Δ*R*^2^	0.09^***^	0.14^***^
*F (df)*	26.92(7)	31.63(7)

*n = 458. The regression coefficients are standardized. Gender: 1 = Male, 0 = Female. Working fulltime: 1 = Fulltime, 0 = Part-time*.

* *p < 0.05*.

** *p < 0.01*.

*** *p < 0.001*.

## Discussion

The current study introduces the construct of work-nonwork boundary management fit and a psychometrically sound measure for it. The perception of work-nonwork boundary management fit is just above average in all four samples (see Table 3, mean scores range from 4.5 to 5.3 on a 7 point scale). Yet, hospital employees on average seem to perceive the highest levels of work-nonwork boundary management fit. A hospital is a work environment with a particular work-life culture and set of practices. Employees that enter this work-environment are often well aware of the prevailing culture and practices and specifically choose that type of work environment. More importantly, all samples display a substantial variance (standard deviations range from 1.14 to 1.36). This reflects a sizable amount of variation in the groups (even within one organization) and thus confirms the relevance of the construct. Moreover, as we put forward, a work environment that fits an employee's boundary management preference (which is reflected in high scores on the proposed scale) is related to various employee attitudes (i.e., job satisfaction, organizational commitment, and turnover intention) and contributes to employees' well-being. Across three independent samples, the work-nonwork boundary management fit scale demonstrated strong internal consistency reliability, construct, and criterion-related validity. In addition, the fourth study demonstrates its unique role in predicting stress and work-life conflict. Considering this particular person-environment fit measure accounts for a significant increase in explained variance, over and above the variance explained by workload and work interfering with nonwork; both determinants that are regularly linked to the experience of stress and role conflict. We thus empirically confirm that work-nonwork boundary management fit is a relevant construct that contributes to employees' subjective well-being and achieving a harmonious work-life interface. In sum, these findings support the basic P-E fit notion that congruence and incongruence are powerful appraisals that employees make about their relationship with a work environment (Edwards and Rothbard, 1999).

### Theoretical contributions

Our findings contribute to the emerging work-life literature in several ways. First, a fitting work environment with regard to boundary management is found to contribute to reduced work-life conflict and thus has the potential to significantly progress the contribution work-family scholars make toward improving employee well-being and work-life balance. Second, considering congruence between boundary management preference and supplies from a needs-supplies fit perspective contributes to a better understanding of how fit affects employees' attitudes and well-being. A fitting work environment with regard to boundary management allows employees to satisfy their boundary management needs, leading to positive attitudes, and improved well-being. Thus, a more complete picture of the role of congruence and how congruence affects employees in the workplace emerges. Third, unlike previous research, work-nonwork boundary management fit taps into employees' cognitive appraisal of congruence between their boundary management needs and perceived supplies provided by the work environment (i.e., perceived fit). This distinction is important because fit may affect attitudes and well-being only if employees consciously experience it (Cable and Derue, 2002; Mackey et al., 2017). Finally, with work-nonwork boundary management fit, researchers can address a central question in work-family research: Why do different employees experience the same work environment as beneficial or harmful for their well-being (Edwards and Rothbard, 1999). Studies in the work-life domain have consistently shown that the same work-life culture and practices affect employees differently (e.g., Rothbard et al., 2005; Foucreault et al., 2016). By introducing the concept of work-nonwork boundary management fit and empirically validating it, we argue that the effectiveness of a certain culture and/or work-life practices depends on employees' experience of congruence between their personal preferences and those set forth in a work environment and must thus be taken into account in theoretical models predicting work-life issues. Overall, this paper makes a crucial step in understanding the importance of perceived congruence in person-environment interactions with regard to the work-life interface.

### Limitations and future research

Although our paper has many strengths, including four diverse employee samples, it is not without limitations. First, all samples stemmed from cross-sectional data (i.e., single-source, self-report data collected at one point in time), creating concerns in terms of common method variance. In addition, we were not able to make any definitive claims of causality. To address the limitations associated with cross-sectional data, we designed the questionnaire in a way to minimize the effects of common method bias (e.g., use of various response formats and protecting respondent anonymity; Podsakoff et al., 2003). An assessment of the extent to which common method bias explained the variance in our constructs showed that common method bias explained <50% of the variance, and therefore did not pose any significant threat to our findings (Podsakoff et al., 2003). Due to the perceptual nature of work-nonwork boundary management fit and employee attitudes and well-being, self-reports are the best (and only) way to capture perceived fit, as it is about the individuals' perception or sense of fitting in. Second, Edwards et al. (2006) distinguished three approaches to the study of perceived P-E fit. The measure of work-nonwork boundary management fit adopts a molar approach and directly measures perceived fit, by asking respondents to rate the fit between themselves and their organization. Although the molar approach has stronger effects on work-related outcomes, the effects may be attributed to subjective evaluations of their organization such that, when employees indicate that they fit the work environment, they are not reporting the result of the cognitive comparison process but instead are effectively saying they are satisfied with their work environment (Edwards et al., 2006). Thus, further research is needed to clarify the meaning of molar perceptions of work-nonwork boundary management fit. Another limitation is that the new construct does not capture one's individual preference separately. Kreiner (2006) findings suggest that having a “neutral” preference toward integration or segmentation is more beneficial for employee well-being than having a strong preference for segmentation or integration, even when the work environment's supplies match this preference. Adding to this, Rothbard et al. (2005) conclude from their findings that (calculated) incongruence in boundary management preference and supplies have less of an effect for employees who prefer integration, compared to employees who prefer segmentation. However, our data in study 4 cannot confirm these notions. Although segmentation preference was found to be positively related to stress (β = 0.15, *p* < 0.01) and work-life conflict (β = 0.19, *p* < 0.001), the interaction between work-nonwork boundary management fit and segmentation preference was not significant in predicting stress (β = −0.07, *ns*.) and work-life conflict (β = −0.03, *ns*.) (additional analyses beyond the main focus of the paper). Therefore, we argue that work-nonwork boundary management fit is important for employee well-being, independent of the employee's boundary management preference. Also the employee's supervisor may play an important role. Allen (2001) states that it is important to disentangle perceptions of supervisor support from perceptions of organizational support with regard to the work-nonwork interface. A family supportive supervisor is “one who is sympathetic to the employee's desire to seek balance between work and family and who engages in efforts to help the employee accommodate his or her work and family responsibilities” (Allen, 2001, p. 417). Thus, despite the perception of work-nonwork boundary management fit with the organization, unsupportive supervisors may undermine the positive effect of fit. Overall, future empirical studies should examine boundary conditions (e.g., boundary management preference) and important moderators (e.g., family supportive supervisor) that affect the relationship between work-nonwork boundary management fit and employee attitudes and well-being.

### Practical implications

Changes in the nature of work, technology, and work demographics have yielded unprecedented potential for organizations to move toward a culture of work-nonwork integration (Rothbard et al., 2005; Rothbard and Ollier-malaterre, 2016). To this end, organizations have adopted numerous policies, practices and norms that allow for work-nonwork integration (e.g., time and space independent working), intended to attract individuals to the organization and help employees manage their multiple roles. Although these policies and practices may increase some employees' satisfaction and performance, our study suggests that one-size-fits-all policies may have drawbacks for other employees who do not perceive fit with their work environment regard to boundary management. The mere availability of certain work-family policies and practices (both formal and informal) creates a work environment that encourages either integration or segmentation of work and personal life for all employees, regardless of their boundary management preference. Organizations are thus facing a tough task. In addition to providing policies and practices that ease the work-life interface, they need to pay attention to the culture and norms they create through these policies and practices. Organizations should recognize the diversity of their employees' needs and preference for segmentation or integration by moving away from one-size-fits all policies and strive to help employees meet their individual needs. Whereas the idea of differentiation among employees and policies is quite common in certain HRM domains (e.g., reward management), the idea of employee diversity in terms of boundary management preference has not yet sufficiently penetrated today's work-family programs and culture. Moreover, organizations should more carefully attract and select employees that fit in the overarching and dominant culture to improve employee well-being and organizational performance.

## Conclusion

Although various studies have empirically confirmed the importance of person-environment congruence with regard to boundary management (e.g., Rothbard et al., 2005; Kreiner, 2006; Chen et al., 2009), a theoretically grounded construct is lacking. The present study developed the construct of work-nonwork boundary management fit that captures *employees' psychological experience of congruence between their personal boundary management preference and the work environment's boundary management supplies*. We empirically confirm that work-nonwork boundary management fit is a relevant construct that has the potential to significantly progress the contribution work-family scholars make toward improving employee well-being and organizational effectiveness. Work-family researchers should therefore pay closer attention to employees' perceived work-nonwork boundary management fit when studying how human resource policies, organizational culture, and supervisor behaviors affect employees' wellbeing, attitudes, and behaviors in the workplace.

## Ethics statement

This study was carried out in accordance with the recommendations of the social and societal ethics committee of the University of Leuven. The protocol was approved by the social and societal ethics committee. All subjects gave written informed consent in accordance with the Declaration of Helsinki.

## Author contributions

YB, RD, and SD developed the research idea and conceptual framework of the article. YB and SD collected the data. YB performed the data analysis and interpreted the results. YB drafted the manuscript. RD and SD provided critical revisions and edited the manuscript. All authors made substantial contributions to the work reported in the article and approved the final version of the manuscript.

### Conflict of interest statement

The authors declare that the research was conducted in the absence of any commercial or financial relationships that could be construed as a potential conflict of interest.
